# Exploring the brachistochrone (shortest-time) path in fire spread

**DOI:** 10.1038/s41598-022-17321-w

**Published:** 2022-08-10

**Authors:** Peiyi Sun, Yanhui Liu, Xinyan Huang

**Affiliations:** Research Centre for Fire Safety Engineering, Department of Building Environment and Energy Engineering, The Hong Kong Polytechnic University, Kowloon, Hong Kong

**Keywords:** Mechanical engineering, Civil engineering

## Abstract

The brachistochrone (shortest-time) curve is the path connecting two points that enables the shortest travel time. This work explores the “brachistochrone path” of fire spread connecting two points at the same altitude and with a fixed path length. The starting and ending points are connected by both thermally thin fuels (thin wires) and thermally thick fuels (PMMA bars). Flame-spread paths of triangular, rectangular, and circular shapes with different heights and inclinations are explored. Results show that having a local maximum flame-spread rate does not result in the shortest overall travel time. For thin-wire paths, the fastest overall-path fire spread occurs, when the upward spread path is vertical, and the path height reaches a maximum, as demonstrated by the theoretical analysis. Differently, for thick PMMA-bar paths, the brachistochrone condition occurs when the path length of the vertical upward spread reaches the maximum, because the upward spread is about ten times faster than the downward spread. This study extends the conventional problem of the fastest fire spread to the shortest-time problem of the whole fire path, and it may help optimize the fuel distribution inside the built environment and estimate available safe egress time in building and wildland fires.

## Introduction

**Figure 1:**
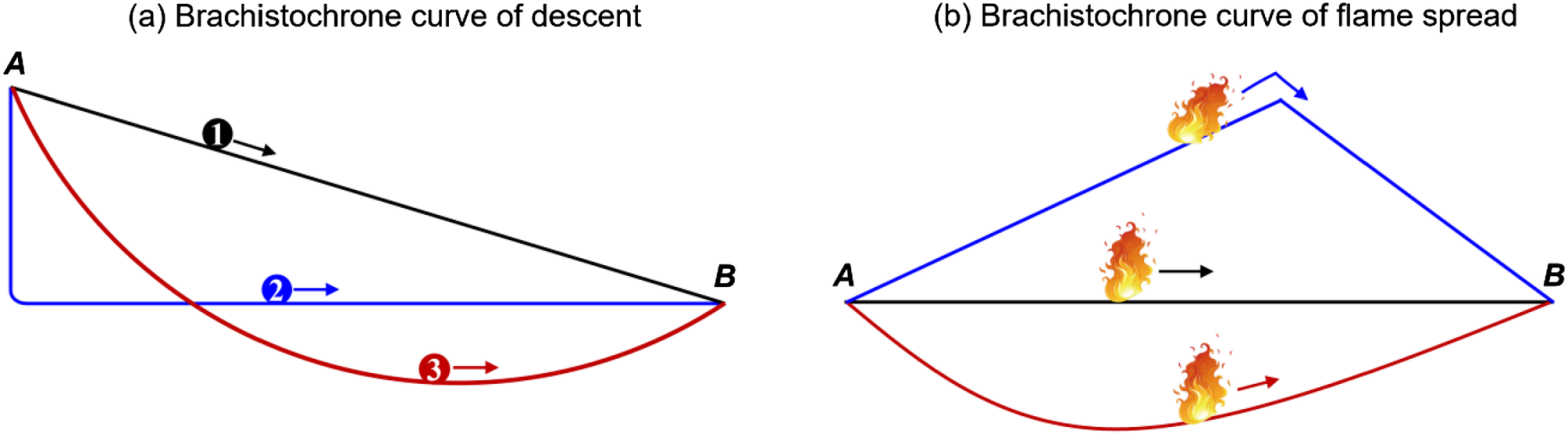
(**a**) The conventional Brachistochrone curve and (**b**) the Brachistochrone curve in fire spread.

The brachistochrone curve in physics and mathematics means the path connecting two points that enables the shortest travel time, where the word ‘brachistochrone’ comes from Ancient Greek ‘shortest time’^1^. It was first proposed by Johann Bernoulli in 1696, who studied the fastest descent (shortest overall travel time) between a point *A* and a lower point *B*. As illustrated in Fig. 1a, *B* is not directly below *A*, on which a bead slides frictionlessly under the influence of a uniform gravitational field to a given endpoint in the shortest time. Bernoulli’s analytical solution showed that the brachistochrone (shortest-time) curve of fastest descent is a tautochrone (or isochrone) curve, different from the shortest-distance curve.

The brachistochrone (shortest-time) curve problem also exists in fire spread, and it is critical to evaluate the overall fire hazard (Fig. 1b). For example, when the flame spreads from village A to village B over the hill, what is the most dangerous hill shape? For a fire spread from room A to room B along a power cable, what kind of cable arrangement will cause the shortest ignition time for room B? Nevertheless, such a shortest-time problem of fire spread has not been systematically studied yet.

Fire spread is essentially a continuous piloted ignition process of the unburnt fuel in the condensed phase^2^. One of the driving forces in fire spread is the buoyant (or the free convective) airflow that is induced by gravity, so it is similar to the ball’s descent process under gravity. However, the fire spread process is much more complex because the conductive and radiant heat transfer from the burning region to the unburned fuel is also important while not affected by buoyancy^3^. Moreover, the inclination of the fuel can also affect the buoyancy flow that changes the contact between flame and fuel surface, thus changing the convective heating and fire spread rate^4–9^.

Based on the relative direction between fire spread and external wind, the fire spread can be divided into concurrent (or forward) spread and opposed spread, and the forward spread is much faster^10,11^. As the buoyant flow is upward, the vertical upward fire spread is generally the fastest^10^, while the vertical downward fire spread could be even slower than the horizontal spread. When a horizontal fuel is inclined upward that exceeds a critical degree, the fire spread rate increases significantly because of the elongation of the flame and the increased heat flux by the contact of flame and fire plume^5,6,8,12^. Such critical inclination angle ranges from 15° to 45°, depending on the fuel type^4,7,13^. In general, the critical angle for thick fuels, like a forest bed^14^ and thick PMMA plate^7,15,16^, is found to be smaller than that for thin fuels like paper^4^ and thin wires^13^.

Most past fire spread studies focused on the rate of spread, particularly the maximum rate, in a fixed inclination with an influence of different environmental factors. However, the overall time of fire spread or the overall path fire spread rate is not well determined. For example, in a hill fire, although the flame has a larger spread rate on the uphill side, the spread rate is much lower on the downhill side. Thus, the fire-spread path with the shortest or the longest residence time is unclear. Especially in real fire scenarios, the fuel components are inhomogeneous, which may result in a sudden increase or decrease of the local fire spread rate. Thus, the average fire spread rate is compared, rather than the fluctuating instantaneous rate. The shortest fire-spread time not only depends on the distribution but also determines the available safe egress time (ASET), which is critical in fire safety design. Thus, there is a knowledge gap.

This study explores the concept of the Brachistochrone (shortest-time) curve in fire spread with small-scale laboratory experiments. Two typical fuels, thermally-thin wire and thermally-thick PMMA bar, are chosen, and the transient and overall fire spread rates are measured. By fixing the overall fire-spread length and the same elevation of start and end points, multiple paths of different shapes are explored. Through varying horizontal span (∆X) and initial orientation ($\theta$) of the fuel, the fastest fire pathway for these wires and PMMA bars were identified and analyzed.

## Experimental methods

### Tested fuels

**Figure 2:**
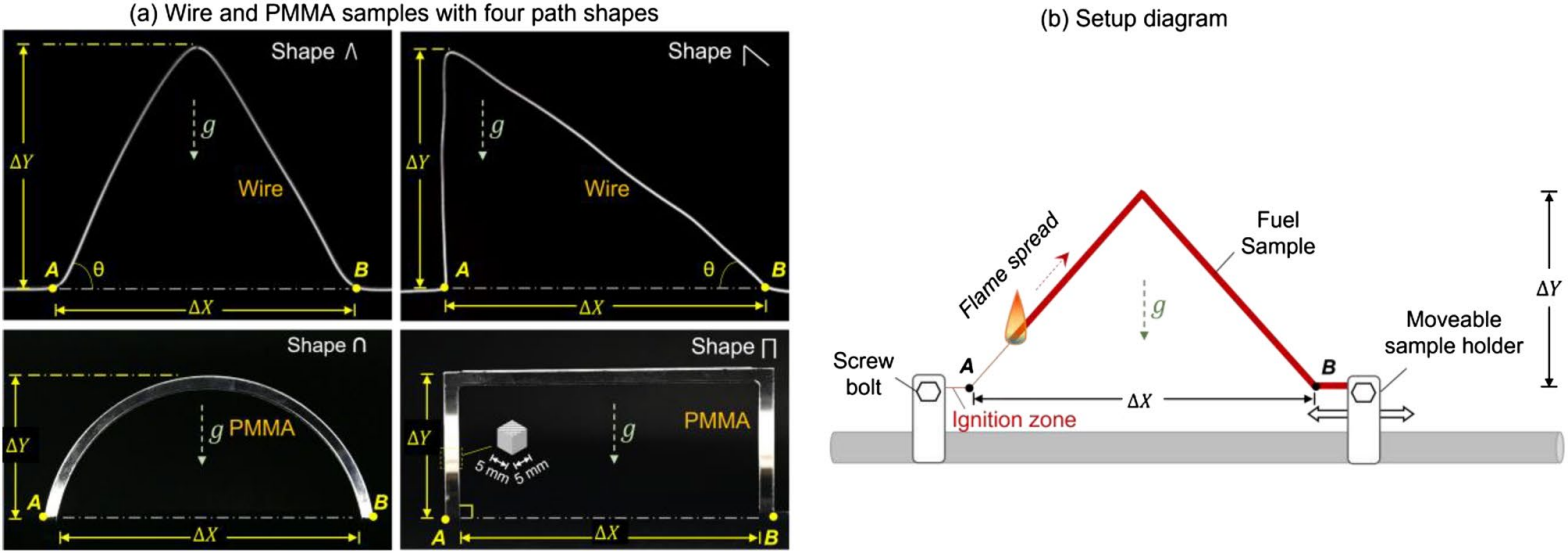
(**a**) Tested thin wires and thick PMMA bar with four curve shapes and (**b**) diagram of the experimental setup.

As the thickness of fuel is the key parameter in fire spread, this work tests both thermally-thin and thermally-thick fuels. To maintain the shape of the thin fuel after the fire spreads over it, a thin wire with a metal core is selected, rather than thin paper stripes. The testing wires are similar to previous studies^9,17–19^. They are made using an iron core and a coating with polyethene (PE), as shown in Fig. 2. This type of wire has good flexibility for making different shapes and is rigid enough to keep the shape during fire spread. Two geometries of wires are tested with the inner core with a diameter (*d*_*c*_) of 0.45 mm and 0.7 mm and the thickness of the PE insulation (*δ*_*p*_) of 0.175 and 0.4 mm, respectively. A thicker insulation layer means more fuels per unit length. Detailed configurations of wires are summarized in Table 1.

**Table 1:** Configuration of thin wires: wire diameter (${d}_{o}$), inner core diameter (${d}_{c}$), insulation thickness (${\delta }_{p}$), and the cross-section area ratio of core to wire (${A}_{c}/{A}_{o}$).

Type	${d}_{o}$ (mm)	${d}_{c}$ (mm)	${\delta }_{p }$ (mm)	${A}_{c}/{A}_{o}$ (%)
I	0.80	0.45	0.175	33
II	1.50	0.70	0.40	22

The thick PMMA bars are laser cut from a cast PMMA plate with 5-mm thickness (Fig. 2a). The width of the sample is also 5 mm, so that cross-section of the PMMA bar is 5 mm × 5 mm square. PMMA with this thickness is thick enough to be considered a thermally thick fuel^20,21^. To avoid the fire spreading along the bottom surface and ensure the experimental repeatability, the PMMA bar is placed above the insulation board of the same shape.

### Tested path curves

Brachistochrone problems can be defined in different ways. For simplicity, in this work, fire spread scenarios and the possible flame-spread path are defined by the following four conditions.

1. The start point (A) and the end point (B) are at the same elevation.
2. The whole path is in the same vertical plane.
3. The total fire spread length is fixed at 20 cm, and
4. The horizontal flame velocity is always positive towards the endpoint (i.e., no backward spread).

In total, five shapes were tested, including three symmetrical shapes (isosceles triangular ∧ or ∨, rectangular ⊓, and arch ◠) and two asymmetrical shapes (lower left triangular  and lower right triangular ), as shown in Fig. 2a.

By adjusting the longitudinal distance or span (∆X), the height (∆Y) and the initial incline angle (*θ*) varied accordingly. The detailed configurations of the four shapes are summarized in Table 2. For all shapes, the minimum span cannot be set to 0; or the endpoint will be ignited during ignition. For the arch shape ◠, the maximum height was 6.4 cm when the shape of the arc was a semicircle, higher than which the flame will spread backwards locally.

**Table 2:** Configurations of path shapes for fire spread.

Pattern type	Span ∆X (cm)	Height ∆Y (cm)	Inclination angle ${\varvec{\theta}}$ ($^\circ$)
Isosceles triangular ∧ or ∨	20	0.0	0 (horizontal)
	18	4.4	26
	16	6.0	37
	14	7.1	46
	12	8.0	53
	10	8.7	60
	8	9.2	66
	6	9.5	73
	4	9.8	78
Rectangular ⊓	14	3.0	90
	10	5.0	
	6	7.0	
	2	9.0	
Arch ◠	18.7	3.0	–
	16.8	4.6	
	15.2	5.5	
	12.8	6.4	
Lower-left and lower-right triangular or	16	3.6	13
	14	5.1	20
	10	7.5	37
	6	9.1	57

The local rate of fire spread (${v}_{f}$) along the path is defined as

1 $${v}_{f}=\frac{dL}{dt}$$

The overall rate of fire spread is defined by the ratio of travel path length (*L* is always equal to 20 cm) to the total spread duration ($\Delta t$) as

2 $${V}_{P}=\frac{L}{\Delta t}$$

Thus, the Brachistochrone curve of fire spread is the path that has the smallest spread duration (${\Delta t}_{min}$)

3 $${\Delta t}_{min}=\frac{L}{{V}_{P,max}}$$

where the largest overall spread rate (${V}_{P,max}$) occurs.

Before the test, the thin wire was bent into the prescribed shape. Then, two ending points of the wire were fixed on the sample holder using a screw bolt (Fig. 2b). The ignition was triggered by a blow torch on the extra horizontal part before the start point (A). For both thin wires and PMMA bar, it only took 3–5 s to ignite a robust flame. Then, the fire spread freely along the path. The fire spread process was recorded by a front-view camera (30 frames per second). The captured video was processed by an in-house MATLAB code to measure the evolution of the local fire spread rate along the path. For each test configuration, at least two repeating tests were conducted to ensure repeatability. All experiments were conducted in a large room without wind.

## Results and discussion

### Effect of concave directions (∧ vs. ∨)

**Figure 3:**
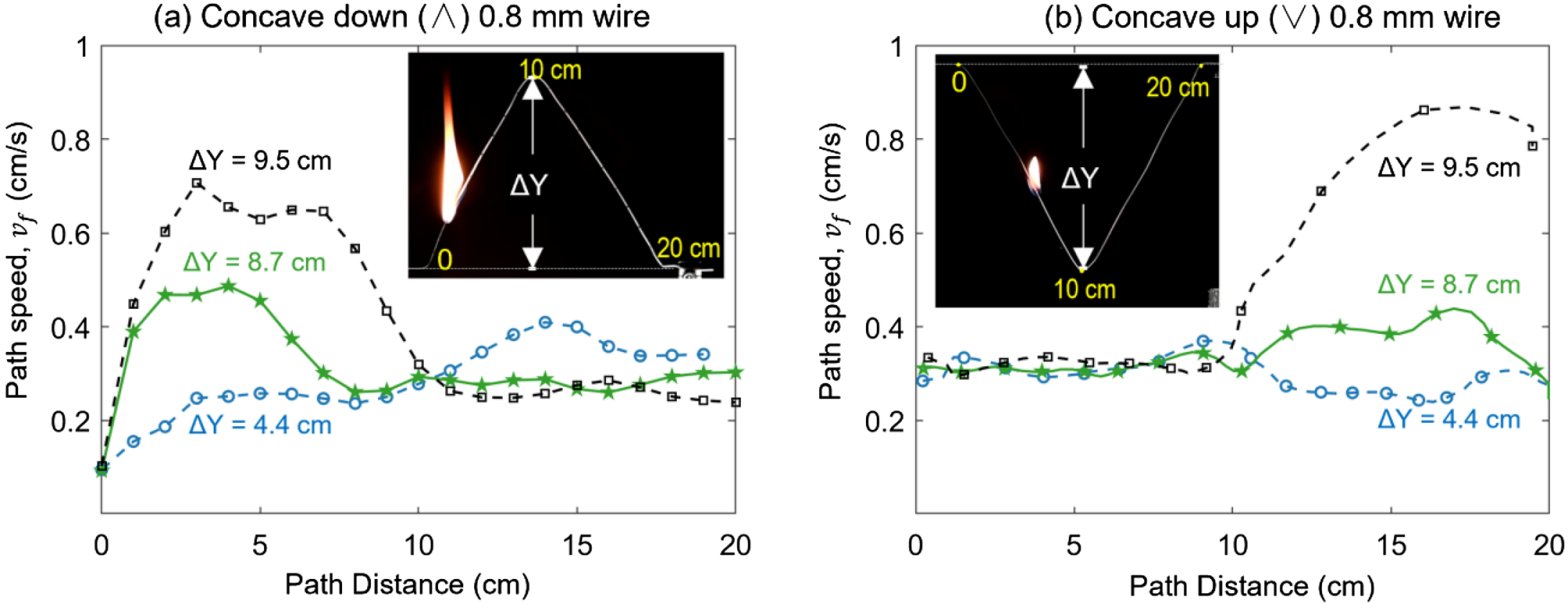
The local speed rate (${v}_{f}$) of (**a**) concave-down “∧” curve and (**b**) concave-up “∨” curve in the isosceles triangle shape.

As the starting and ending points have the same elevation, the burning process would either only include the horizontal fire spread process (with the largest span ∆X) or involve both upward and downward fire spread. For each shape, it has two concave directions, first upward and then downward (i.e., concave down “∧”) and first downward and then upward (i.e., concave up “∨”). Figure 3 compares the local flame-spread rate (${v}_{f}$) along a pair of isosceles triangular paths (a) concave down and (b) concave up (see Video S1).

For the wire in a concave-down “∧” curve in Fig. 3a, the fire spreads upwardly first, so that the path speed increases dramatically within the first 10-cm path. After the turning point of 10 cm, the fire spreads downward: the path speed is almost constant in this stage. As the path height (∆Y) increases, the span reduces correspondingly, and the inclination is more towards vertical upward. Thus, the maximum local fire spread rate (${v}_{f, max}$) increases.

The concave-up “∨” curve in Fig. 3b shows an opposite trend in the local fire spread rate. The fire spreads down first, showing a near-constant fire spread in the first 10 cm. After the turning point, the upward fire spread is assisted by the buoyancy flow, resulting in the acceleration of the fire spread.

**Figure 4:**
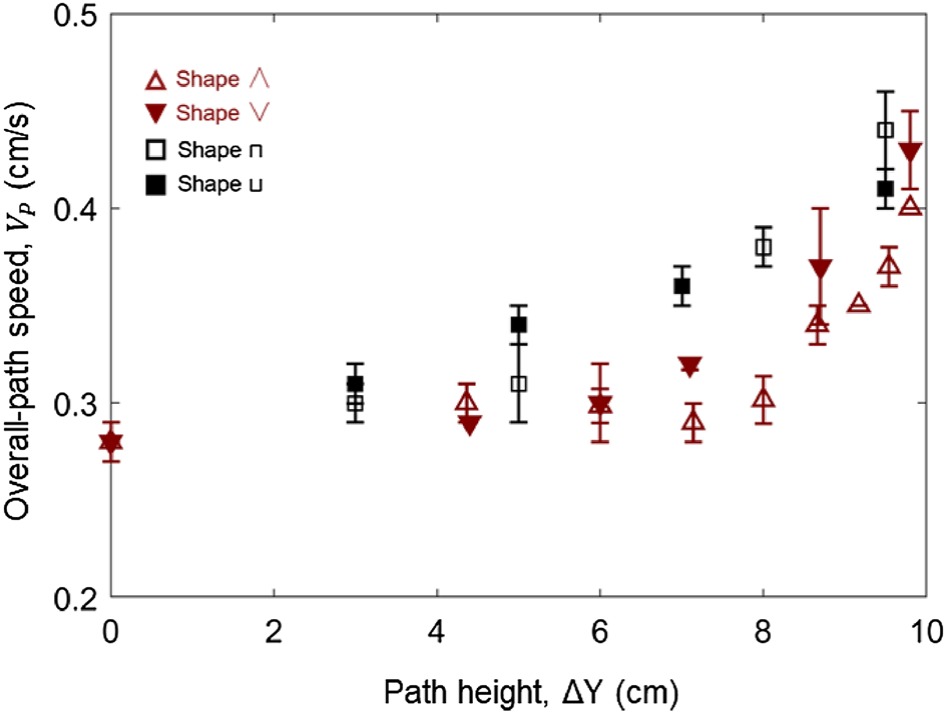
The overall-path speed (${V}_{P}$) vs. concave directions for the 0.8-mm wires.

Figure 4 further compares the overall-path speed (${V}_{P}$) between concave-down and concave-up paths of different shapes (∧ vs ∨ and ⊓ vs ⊔). Essentially, the differences in ${V}_{P}$, caused by the direction of concavity, are within the error bars (uncertainty) of the repeated tests. As similar trends are also found in other tested fuels, we can conclude that the effect of concave direction is negligible. Therefore, in the following text, only the concave-down curves are analyzed.

### Effect of path inclination

**Figure 5:**
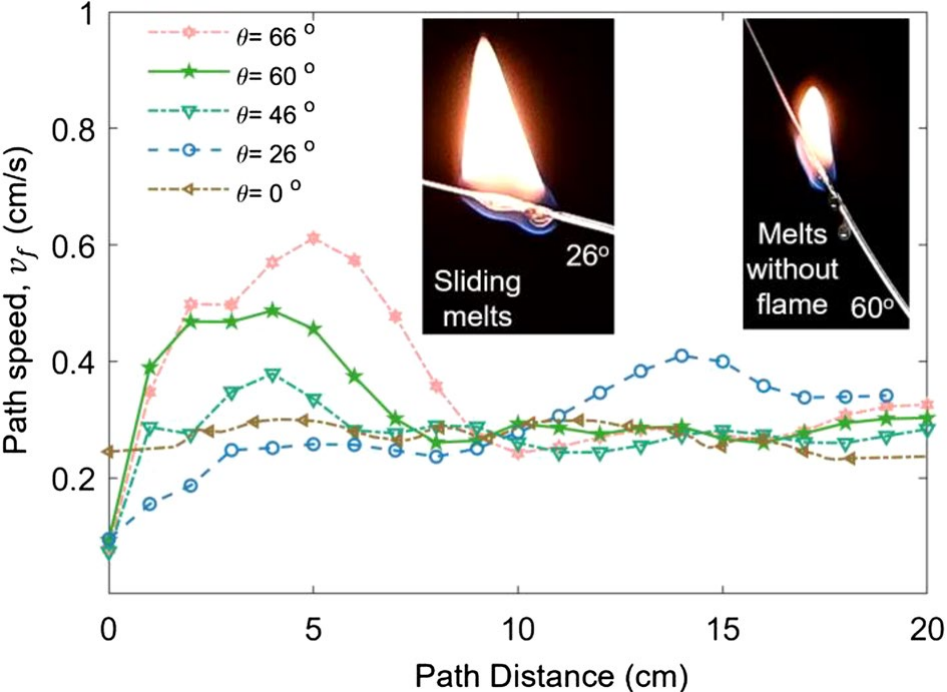
The local flame speed (${v}_{f}$) along 0.8-mm thin wire with different inclined angles.

Past studies^4–9,13–15^ have shown that the inclination angle has a significant effect on the fire spread rate, regardless of the fuel thickness. In this study, the inclination angle ($\theta$) is identified as the uprising angle at the start point. For isosceles triangular curves, a larger inclination angle represents the larger path height change (∆Y) and smaller horizontal span (∆X). Figure 5 plots the local path speed ($v_{f}$) along the 0.8-mm thin wire under different inclinations (Video S2). For the first half upward, the spread rate generally increases with the inclination angle, especially if the inclination is larger than the critical angle of ~ 50°.

Nevertheless, there is a special path with the 26° inclination. It has the lowest upward fire spread rate, even lower than the horizontal base case (0°). On the contrary, in the downward fire spread stage, it has the largest fire spread rate of the whole path. This is mainly caused by the melting of the thermoplastic that accelerates the downward spread and decelerates the upward spread^22^. With a small incline angle, the melting plastic will slide down along the wire. In the upward spread, the dripping removes fuel in the burning zone, reducing the flame size and slowing down the fire spread. In the downward spread, the melts slide down with a flame to preheat downward and accelerate the fire spread. Therefore, the preheating process is not only dominated by the nearby flame but also the movement of melting fuel. With the increasing inclination angle, the effect of dripping flow declines^23^, as the melts no longer carry a flame (see Fig. 5). Similar phenomena are also observed for the 1.5-mm thin wire.

**Figure 6:**
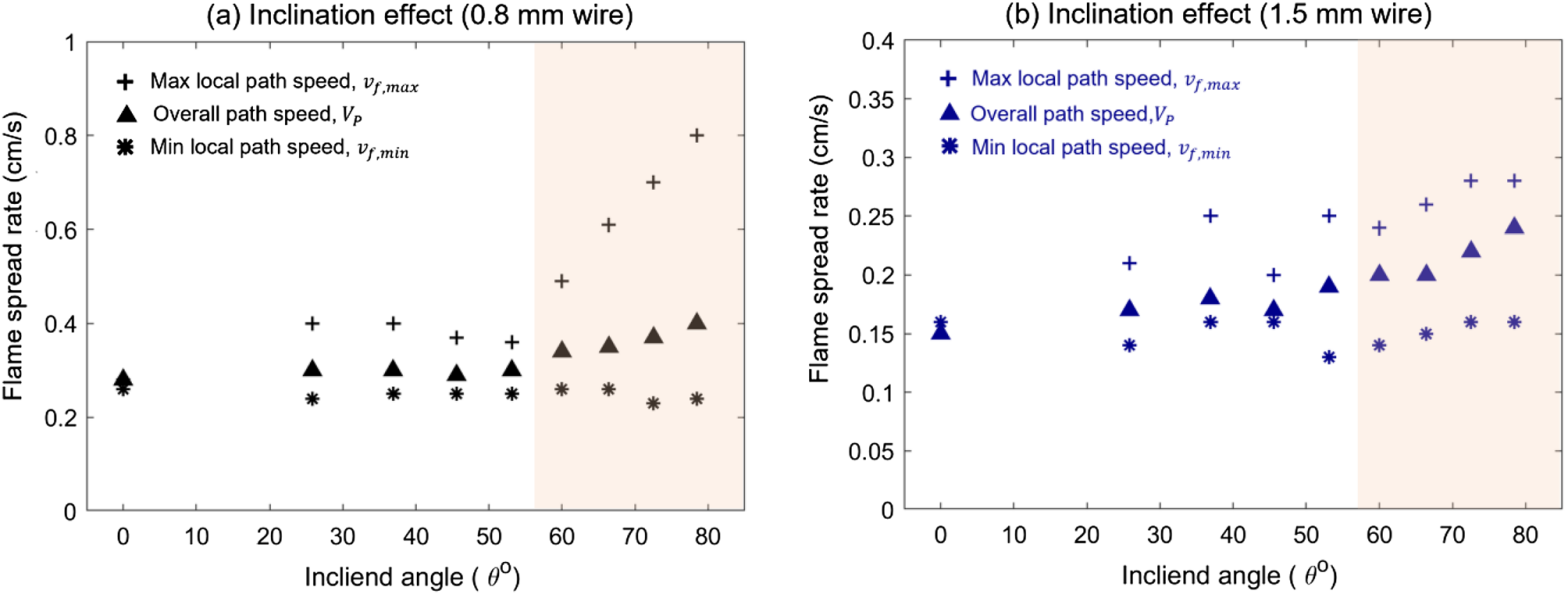
The inclination effect on characteristic spread rates for typical thin fuels (**a**) 0.8-mm thin wire and (**b**) 1.5-mm thin wire.

The influences of inclination angle on the overall fire spread rate ($V_{P}$), and local maximum ($v_{{{\text{f}},\max }}$) and minimum ($v_{{{\text{f}},\min }}$) are summarized in Fig. 6a. When the inclination angle is lower than 50°, the overall spread rate is almost insensitive to the inclination angle. At $\theta$ > 50°, the overall-path fire spread rate increases monotonically. A similar transition angle also can be observed for the wire with a 1.5 mm diameter in Fig. 6b. As the thickness of the fuel increases, the overall fire spread rate decreases as well. Note that the critical angle for overall fire spread rate for fuel curve is slightly larger than those (15°–45°) for the straight fuel in the literature^4,7,13^.

Figure 6 also shows that the maximum local speed (${v}_{f, max}$) is only 20% larger than the overall value, while the minimum is only 15% smaller than the overall value. Similar to the trend of overall speed, the maximum local speed (${v}_{f, max}$) also increases with the inclined angle when the angle exceeds 50°. In contrast, the minimum local spread rate (${v}_{f, min}$) is insensitive to the inclined angle. The maximum local speed reflects the effect of upward buoyancy flow, so it increases with the inclination angle, while neither the effect of buoyancy flow nor melting flow can significantly change the downward fire spread.

### Effect of path shape

**Figure 7:**
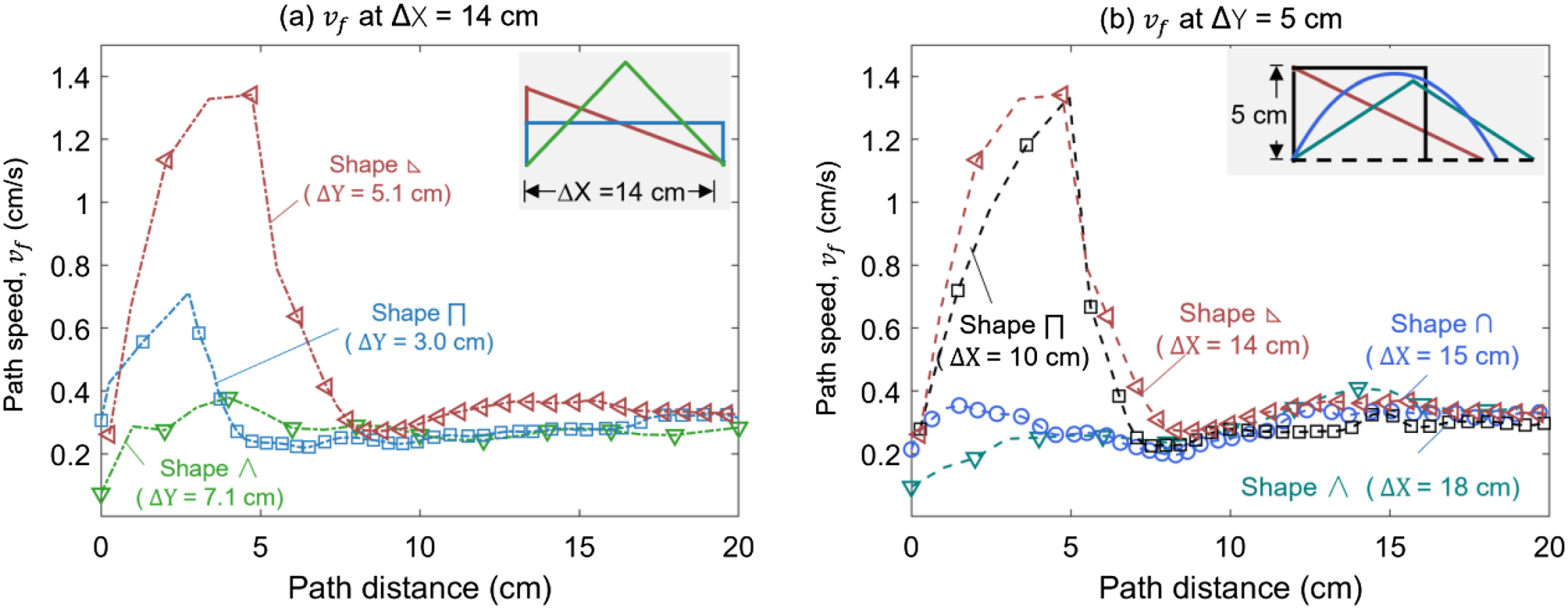
The path speed (${v}_{f}$) for different path shapes (**a**) under the same horizontal span (∆X = 14 cm) and (**b**) under the similar path height (∆Y = 5 cm).

Figure 7a shows the local fire spread rate along with the 20-cm thin wires with three shapes, where the horizontal span is fixed to ∆X = 14 cm. It is apparent that the flame has the largest local spread rate over the lower-left triangular shape . The tallest isosceles triangular shape ∧ should have the largest local speed because of the longest upward fire spread distance, but it shows the lowest local fire spread rate (Fig. 7a). The fire spread over thin fuel reaches a semi-steady state quickly, and it does not accelerate much with the height. Thus, the highest path of thin fuel does not necessarily result in the largest spread rate. Comparatively, the inclination angle is more important, and the vertical upward spread has the local maximum speed. Thus, the path speeds of the lower-left triangular shape  and rectangular shape ⊓ speed faster than the isosceles triangle in the upward spread stage. Because the downward fire spread is insensitive to the shape, the overall fire spread rate of thin fuel highly depends on the maximum upward spread rate.

When the path height is fixed around ∆Y = 5 cm, Fig. 7b compares the flame path speed over four shapes with different horizontal spans. Similarly, the path shape has a significant influence on the upward fire spread rate but has less impact on the downward spread rate. It is evident that the upward fire spreads faster over the shapes with smaller horizontal spans (rectangular ⊓ and lower left triangular ) because the smaller horizontal span has a larger inclined angle to enhance the buoyancy-driven upward fire spread. Thus, for thin fuels, the impact of path shape is primarily reflected by the upward spread path.

**Figure 8:**
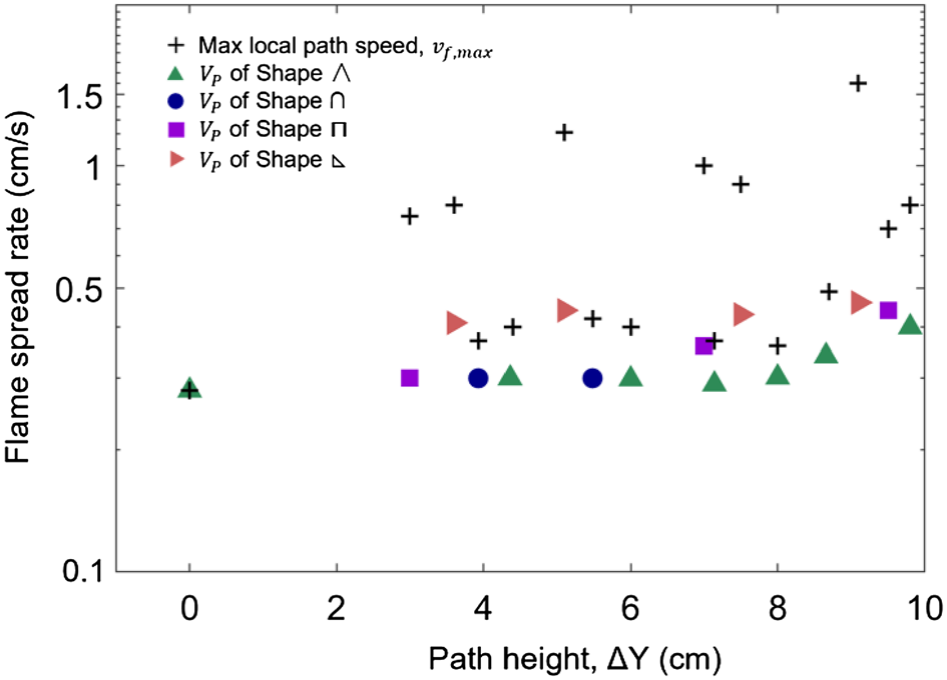
The overall fire-spread path speed (${V}_{P}$) vs. ${v}_{f, max}$ of all path shapes.

Figure 8 further summarizes the fastest spread paths at different heights, as well as the corresponding maximum overall spread rate ($V_{{{\text{P}},\max }}$) and local maximum speed (${v}_{f, max}$) of all shapes. It is verified that the maximum overall path speed is significantly affected by the shape. More importantly, the maximum local fire spread rate is irrelevant to the maximum overall path speed. For example, the maximum local rate for rectangular path (⊓) occurs at ∆Y = 7.5 cm, whereas its overall path speed is not the largest there.

### Effect of fuel thickness

**Figure 9:**
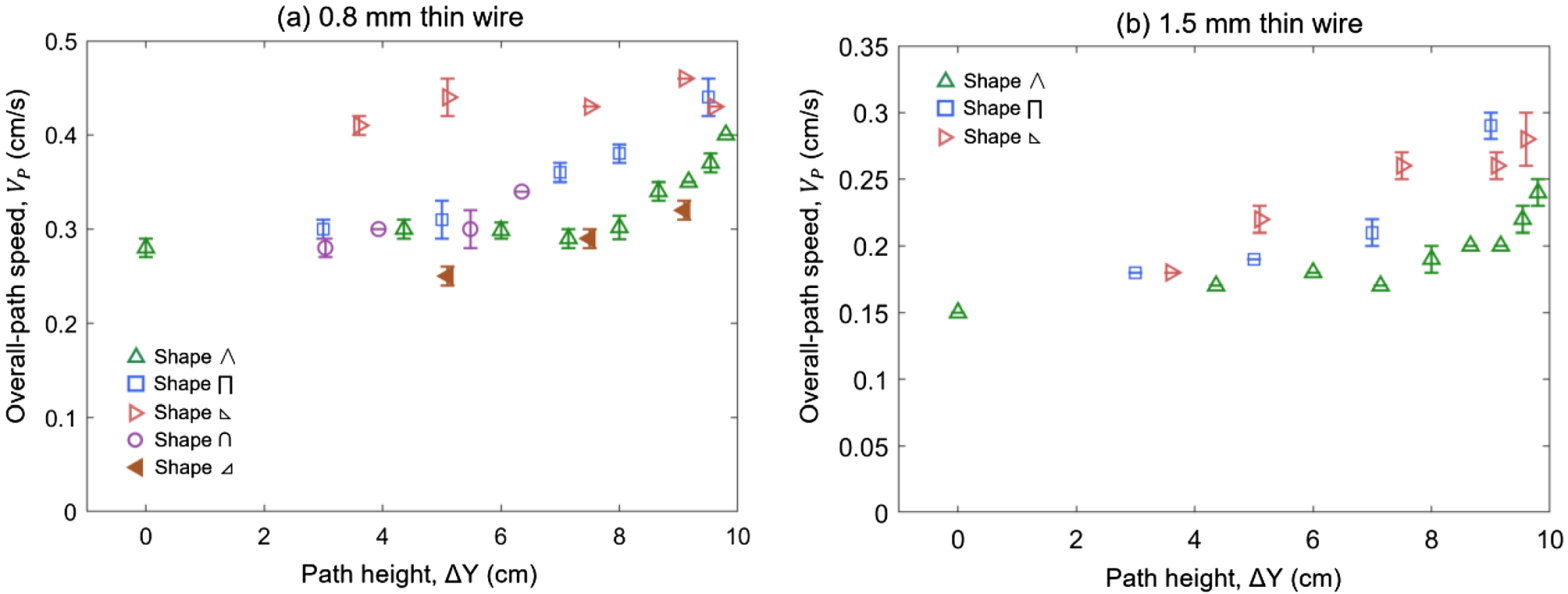
The overall fire spread rate (${V}_{P}$) for all path shapes varied with the path height for (**a**) 0.8-mm thin wire and (**b**) 1.5-mm thin wire.

The fuels with different thicknesses were tested in this study. The thin wires with two dimensions represent the thermally thin fuel, and the thick PMMA samples represent the thick fuel. As the discussion in Sect “Effect of path shape”, the overall fire spread speed has a great variation with the path shapes. Figure 9 plots the overall fire spread rate of all shapes for two thin wires (Video S3).

The lower left triangular shape  is more sensitive with the maximum height, and it shows the shortest fire spread route. The 1.5 mm thin wire shows a similar trend (Fig. 9). The sensitivity of the overall fire spread rate to the height variation for three patterns follows: lower left triangle > rectangular shape > isosceles triangle shape. As expected, for a thin fuel, with the increasing of the fuel thickness, the overall fire spread rate reduces correspondingly (Fig. 9a,b).

**Figure 10:**
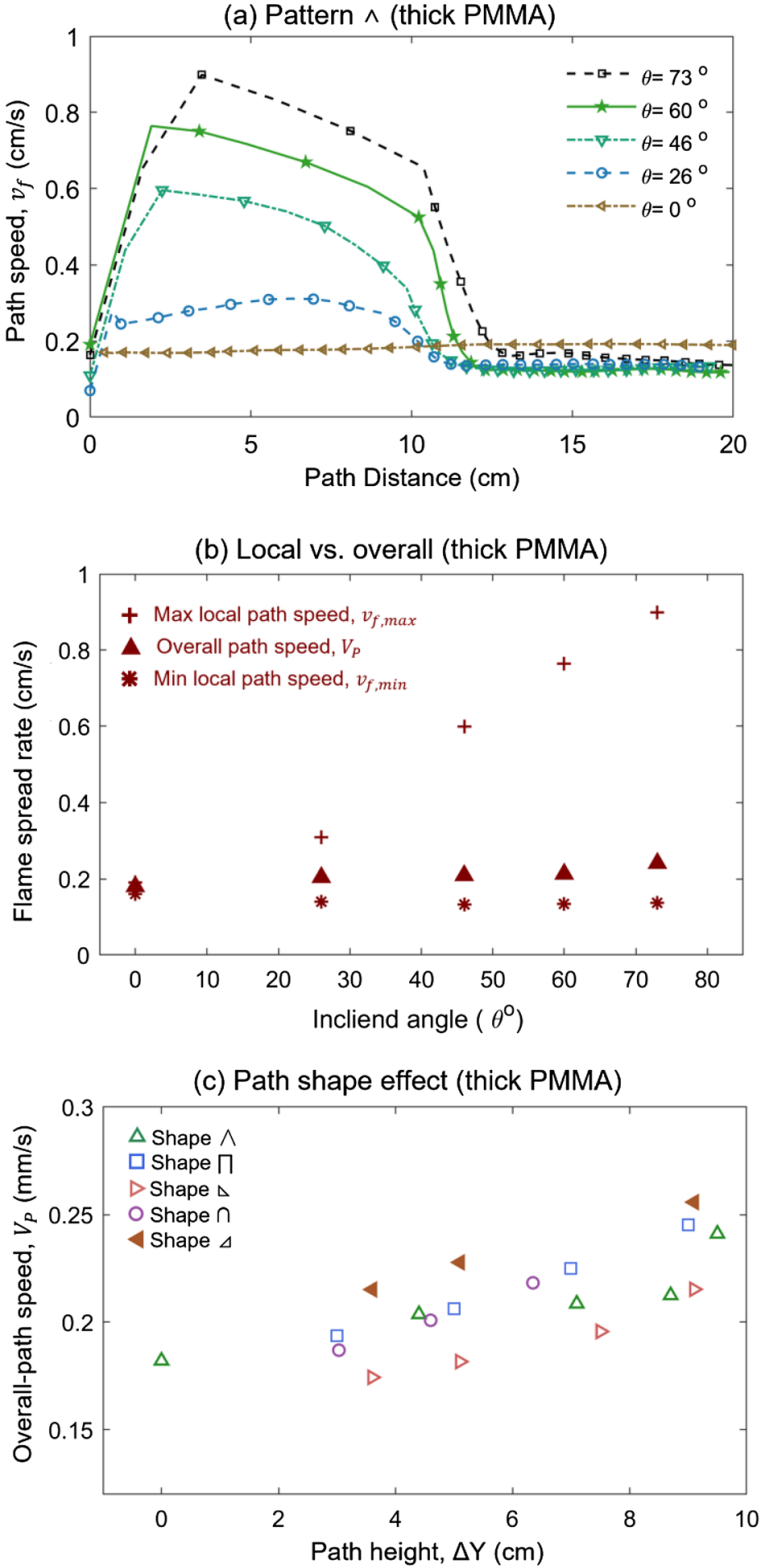
Fire spread rate on 5-mm thick PMMA bar, (**a**) local fire speed (${v}_{f}$) with different inclined angles, (**b**) overall fire spread rate (${V}_{P}$) vs. local maximum (${v}_{f, max}$) and minimum (${v}_{f, min}$), and (**c**) overall fire spread rate (${V}_{P}$) all path shapes.

For the thick PMMA bar, the maximum local speed increases significantly with the inclined angle (see Fig. 10a and Video S4). Nevertheless, the overall path speed and the minimum local speed are almost comparable and unchanged with the varied inclined angle (Fig. 10b), different from the thin wires in Fig. 6a,b. Also, for the thick PMMA, the lower-left triangular  has the lowest fire spread rate, while the lower-right triangular  has a much faster overall spread rate (Fig. 10c). Moreover, there is a big difference between thermally thin and thermally thick fuels. For the thin fuel, the strategy of achieving the fastest spread route is offered by the greater inclination angle at the beginning side. However, for thick fuel, the key parameter is guaranteeing the longer upward fire spread pathway.

**Figure 11:**
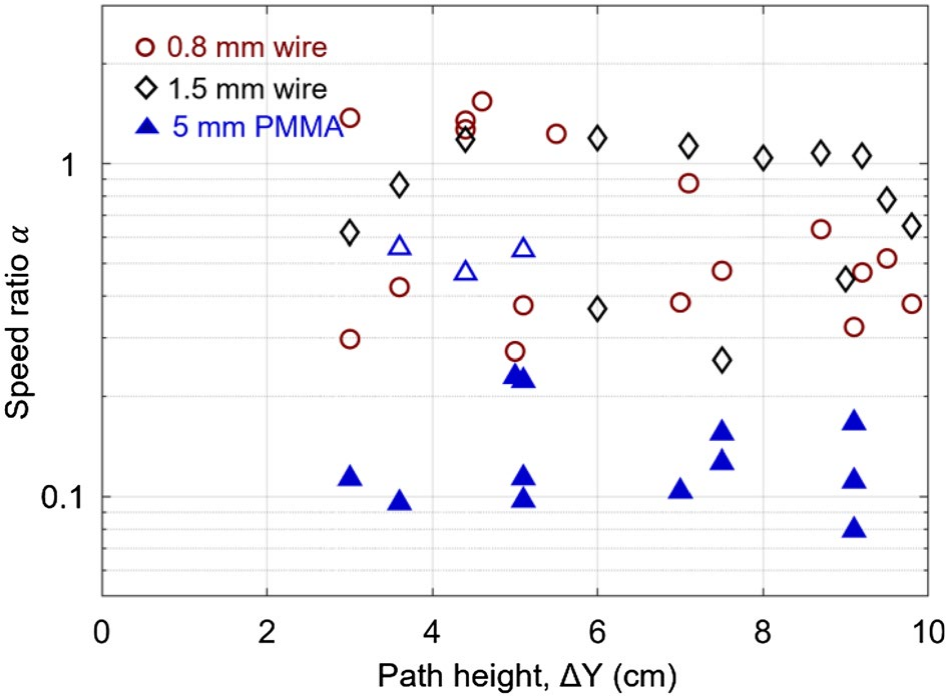
The average speed ratio $(\alpha )$ varied with the path height in three fuel thicknesses.

Another difference caused by the fuel thickness is in the average speed ratio (Fig. 11). Herein, we define the ratio of the average downward speed to the upward speed of the path as

4 $$\alpha = \frac{{V_{d} }}{{V_{u} }} = \left\{ {\begin{array}{*{20}l} {0.3{\text{--}}1.2\;({\text{thin wire}})} \hfill \\ {0.06{\text{--}}0.2\;({\text{thick PMMA}})} \hfill \\ \end{array} } \right.$$

Overall, the speed ratio of thin wires is much larger than that of thick PMMA. For thin wire, some speed ratios would be larger than 1 (${V}_{u}<{V}_{d}$), where the wire has a smaller inclination angle, and the melting flow becomes important. Further increasing the wire inclination angle until exceeding the critical inclination angle (50°), the upward flame speed rate increases dramatically, driven by the buoyancy flow and resulting in a smaller speed ratio.

Similarly, for the PMMA bar, when the inclined angle is smaller than the critical angle of 20°, the upward fire spread rate is comparable to the downward spread rate, showing the relatively large ratio (marked as the hollow triangle in Fig. 11). Different from the thin fuel, the upward spread speed increases significantly (${V}_{u}\gg {V}_{d}$) with the increasing inclination angle, showing the lower average speed ratio. The different sensitivity of $\alpha$ to different fuel thicknesses should be the critical reason for different brachistochrone fire-spread paths between thin and thick fuels.

### Brachistochrone (shortest-time) analysis

Mathematically, the overall fire-spread duration ($\Delta t$) is equivalent to the sum of upward-spread time (${\Delta t}_{u}$) and downward-spread time (${\Delta t}_{d}$) as

5 $$\Delta t={\Delta t}_{u}+{\Delta t}_{d}$$

The total length of the path includes the upward path length (${L}_{u}$) and downward path length (${L}_{d}$) that is fixed in the current problem, i.e., $L={L}_{u}+{L}_{d}=20$ cm. By introducing the overall-path (average) fire spread rates in Eq. (2) for both upward and downward paths, the total duration can be further expressed as

6 $$\Delta t=\frac{{L}_{u}}{{V}_{u}}+ \frac{{L}_{d}}{{V}_{d}}{=L}_{u}(\frac{1}{{V}_{u}}- \frac{1}{{V}_{d}})+\frac{L}{{V}_{d}}$$

where ${V}_{u}$ is the average rate for upward fire spread, and ${V}_{d}$ is the average rate for the downward spread. The average spread rate depends on the path shape and fuel type, as demonstrated by the test data above.

For the thermally thick PMMA bars, the upward fire spread could be 10 times faster than the downward spread, that is, ${V}_{u}\gg {V}_{d}$ or $\alpha <0.2$ (see Fig. 11). Thus, the time spent in the upward spread (${\Delta t}_{u}$) is negligible, and the total time can be approximated as

7 $$\Delta t\approx \frac{{L}_{d}}{{V}_{d}}$$

Because the downward spread rate (${V}_{d}$) is insensitive to the shape (see Fig. 10a), the overall fire spread duration ($\Delta t$) reaches the minimum, as the downward spread length ${L}_{d}$ is the smallest, or the upward spread length is the longest. Therefore, the brachistochrone path for fire spread over the thick PMMA bars corresponds to the shape of lower right triangular , which has the longest upward spread length, given a path height ($\Delta {\text{Y}}$). This analysis agrees well with the experimental results in Fig. 10c. Such a conclusion is also expected to be valid for fire spread on other thick fuels.

For thermally thin wires, the value of ${V}_{d}$ is only slightly smaller than ${V}_{u}$ (see Figs. 3a, 5, 11). Considering that the ratio $\alpha ={V}_{d}/{V}_{u}$ >0.3, the total time can be re-arranged as

8 $$\Delta t=\frac{L}{{V}_{d}} \left[1-\frac{{L}_{u}}{L}(1-\alpha )\right]$$

Because the downward spread rate (${V}_{d}$) is almost a constant, the value of $\Delta t$ decreases as ${L}_{u}$ increases and $\alpha$ decreases. To decrease the value of $\alpha ={V}_{d}/{V}_{u}$, the upward fire spread rate (${V}_{u}$) should reach the maximum, suggesting that the path should include the vertical upward fire spread, i.e., rectangular ⊓ and triangular . At the same time, the value of ${L}_{u}$ in the upward fire spread is the same as path height ($L_{u} = \Delta {\text{Y}}$), so that increasing the path height can reduce the total duration.

In summary, the shortest-time path for fire spread over thin wires should correspond to the shape of lower left triangular  with the maximum path height, agreeing with the experimental results in Fig. 9. Such a conclusion can also be extended to fire spread over other thin fuels. Different brachistochrone paths are found for thin and thick fuels. It is because the ratio of downward fire spread rate to upward fire spread rate ($\alpha = V_{d} /V_{u}$) decreases significantly with increasing fuel thickness. To further validate the proposed brachistochrone paths, the fire spread over more fuel-bed types of larger scales can be tested in future work.

**Figure 12:**
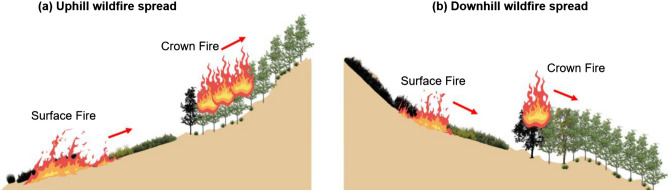
Illustration of wildfire spread: (**a**) uphill fire spread and (**b**) downhill fire spread (copyright: authors).

The finds in this work may have important implications for wildland fire spread behaviors, where fire spreads over hills of different slopes with different wildland fuels. Depending on the location of ignition and slope of the landscape, wildfire can spread uphill (i.e., the head fire) or downhill (i.e., the back fire), as illustrated in Fig. 12. For the spread of a typical surface fire, the primary fuel is the shrubs, grass, or dead fuel litter layer that are typically thermally thin. For the spread of the crown fire, the tree crow is often thermally thick, although leaves and small twigs inside the crown could be thin.

The spread of wildfire always follows or is dominated by the fuel path of the shortest spread time, and the leading edge of flame is its fastest-spread front (i.e., a typical brachistochrone problem). Thus, it is possible to find the fastest fire-spread path and the overall wildfire spread time, based on the landscape and fuel type. Although a complex numerical wildfire model can reveal more details, it needs high-resolution inputs of environment and fuel that are not often available. Based on the principle discovered in this work, the potential safe evacuation path and time for local residents can be quickly estimated. It can help the fire safety design of the wildland-urban interface and plan for the emergency evacuation in case of wildfire.

## Conclusions

This work explores the brachistochrone (shortest-time) problem in fire spread connecting two points at the same altitude and with the fixed path length and fuel load. Experimental results show that the concave direction has a negligible effect on the overall fire spread rate. Also, the maximum local flame-spread rate does not result in the shortest overall travel time. The inclination angle, shape, and path height are found to significantly affect the overall fire spread rate.

For thin-wire paths, the fastest overall-path fire spread occurs when the upward spread path is vertical (i.e., the shape of lower left triangular ), and the path height reaches the maximum. Comparatively, for thick PMMA-bar paths, the brachistochrone condition occurs when the path length of the upward fire spread reaches the maximum (i.e., lower right triangular ). The proposed theoretical analysis successfully explains the experimental observation and reveals that different brachistochrone conditions for thick and thin fuels are caused by different ratios of downward to upward fire spread rate. This study extends the conventional problem of the fastest fire spread to the shortest-time problem of the whole fire path. This may help optimize the fuel distribution inside the building and estimate the available safe egress time in building and wildland fire.

## Supplementary Information

Supplementary Video S1.

Supplementary Video S2.

Supplementary Video S3.

Supplementary Video S4.

## Data Availability

The datasets used and/or analyzed during the current study available from the corresponding author on reasonable request.
